# Recognition of Fibrotic Infarct Density by the Pattern of Local Systolic-Diastolic Myocardial Electrical Impedance

**DOI:** 10.3389/fphys.2016.00389

**Published:** 2016-08-31

**Authors:** Gerard Amorós-Figueras, Esther Jorge, Tomás García-Sánchez, Ramón Bragós, Javier Rosell-Ferrer, Juan Cinca

**Affiliations:** ^1^Department of Cardiology, Hospital de la Santa Creu i Sant Pau, Institut d'Investigació Biomèdica - Sant Pau, Universitat Autònoma de BarcelonaBarcelona, Spain; ^2^Electronic and Biomedical Instrumentation Group, Department of Electronics Engineering, Universitat Politècnica de CatalunyaBarcelona, Spain

**Keywords:** healed myocardial infarction, myocardial electrical impedance, hemodynamics, novel bioimpedance device, swine

## Abstract

Myocardial electrical impedance is a biophysical property of the heart that is influenced by the intrinsic structural characteristics of the tissue. Therefore, the structural derangements elicited in a chronic myocardial infarction should cause specific changes in the local systolic-diastolic myocardial impedance, but this is not known. This study aimed to characterize the local changes of systolic-diastolic myocardial impedance in a healed myocardial infarction model. Six pigs were successfully submitted to 150 min of left anterior descending (LAD) coronary artery occlusion followed by reperfusion. 4 weeks later, myocardial impedance spectroscopy (1–1000 kHz) was measured at different infarction sites. The electrocardiogram, left ventricular (LV) pressure, LV dP/dt, and aortic blood flow (ABF) were also recorded. A total of 59 LV tissue samples were obtained and histopathological studies were performed to quantify the percentage of fibrosis. Samples were categorized as normal myocardium (<10% fibrosis), heterogeneous scar (10–50%) and dense scar (>50%). Resistivity of normal myocardium depicted phasic changes during the cardiac cycle and its amplitude markedly decreased in dense scar (18 ± 2 Ω·cm vs. 10 ± 1 Ω·cm, at 41 kHz; *P* < 0.001, respectively). The mean phasic resistivity decreased progressively from normal to heterogeneous and dense scar regions (285 ± 10 Ω·cm, 225 ± 25 Ω·cm, and 162 ± 6 Ω·cm, at 41 kHz; *P* < 0.001 respectively). Moreover, myocardial resistivity and phase angle correlated significantly with the degree of local fibrosis (resistivity: *r* = 0.86 at 1 kHz, *P* < 0.001; phase angle: *r* = 0.84 at 41 kHz, *P* < 0.001). Myocardial infarcted regions with greater fibrotic content show lower mean impedance values and more depressed systolic-diastolic dynamic impedance changes. In conclusion, this study reveals that differences in the degree of myocardial fibrosis can be detected *in vivo* by local measurement of phasic systolic-diastolic bioimpedance spectrum. Once this new bioimpedance method could be used via a catheter-based device, it would be of potential clinical applicability for the recognition of fibrotic tissue to guide the ablation of atrial or ventricular arrhythmias.

## Introduction

Myocardial electrical impedance is a biophysical marker of the state of integrity of the myocardial tissue (Sperelakis and Hoshiko, 1961). Early studies conducted in experimental animal models have consistently shown that acute myocardial ischemia induced by coronary artery ligation increased dramatically the myocardial impedance (Cinca et al., 1998; Padilla et al., 2003; Rodriguez-Sinovas et al., 2005). In contrast, during the healing over process the necrotic scar turns to lower than normal impedance values (Fallert et al., 1993; Wolf et al., 2001; Salazar et al., 2004). In these studies, the technique of measuring the changes in myocardial impedance did not allow to delineate the sequential impedance variations elicited during the systole and diastole phases of the cardiac cycle because of the long time required for the whole impedance spectrum acquisition (Gersing, 1998; Casas et al., 1999; Warren et al., 2000; Cinca et al., 2008). Quite recently, the use of refined fast broadband electrical impedance spectroscopy (EIS; Sanchez et al., 2011) allowed to describe the cyclic pattern of systolic-diastolic impedance changes in a swine model of acute myocardial ischemia (Jorge et al., 2015). As compared with the classical technique, the new EIS-based method permitted a prompt detection of local myocardial ischemia based on the occurrence of specific changes in the phasic systolic-diastolic impedance curve. These were characterized by an holosystolic resistivity rise leading to a bell-shaped impedance waveform and a reduction of the area under the left ventricular (LV) pressure-impedance curve. In that study, the holosystolic impedance rise maintained a temporal relationship with the local mechanical dyskinesis induced in the ischemic region, thus it may emerge as a new indicator (surrogate) of early acute myocardial ischemia. During the healing-over process the acute ischemic myocardium suffers a profound structural and functional remodeling, with collagen deposition and fibroblasts proliferation. These structural alterations will affect the pattern of systolic-diastolic impedance of the infarct scar region, but this has not yet been investigated.

This study aimed to characterize the phasic (systolic-diastolic) changes of myocardial resistivity in the *in situ* swine heart with 1 month old healed myocardial infarction by using fast broadband EIS. Specifically, we assessed how myocardial resistivity is influenced by the amount of fibrotic content of the infarct scar.

## Materials and methods

The study protocol was approved by the Animal Care and Use Committee of our institution and conformed to the *Guide for the Care and Use of Laboratory Animals*: Eighth Edition (National Research Council. Washington, DC: The National Academies Press, 2010).

### Study protocol

The study began after stabilization of the level of anesthesia and the hemodynamic parameters. Then, the ECG, LVP, ABF and impedance signals were recorded during 4 s at each site. In each pig, we explored 8–11 myocardial impedance sites spaced 10 mm apart, beginning at the healthy region and extending toward the infarcted area. At the end of the study the animals were euthanized by administration of an intravenous KCl overdose and the heart was removed.

### Experimental preparation

Eight female domestic swine (Landrace-Large White cross) weighing 33 ± 2 kg were submitted to two interventions spaced 30 days. The first intervention aimed at inducing an acute myocardial infarction. Animals received 400 mg oral amiodarone and 500 mg aspirin from 2 days before to 7 days after infarct induction. They were premedicated (midazolam 0.6 mg/kg and ketamine 12 mg/kg), anesthesized (propofol 2–4 mg/kg), and maintained with sevoflurane inhalation (2.5–3.5%). After endotracheal intubation animals were mechanically ventilated. Analgesia was maintained with a perfusion of remifentanil (0.2 μg·kg^−1^·min^−1^). Continuous infusion of lidocaine at a rate of 1 mg/kg/h (Lidocaina Braun. B. Braun Medical, Barcelona, Spain) was used through the procedure. Systemic sodium heparin was injected IV (150 UI/kg) 5 min prior to percutaneous sheath placement.

Under aseptic conditions, a femoral arterial access was established using the Seldinger technique and a 7F introducer sheath (Cordis; Miami, FL) was placed percutaneously into the femoral artery. To better mimic the clinical human scenario of a reperfused infarction, a coronary artery occlusion-reperfusion was performed. The infarction created with this models is characterized histologically by surviving strands of myocardium interwoven with fibrotic scar. A small modification of the Sasano model was used (Sasano et al., 2006). Briefly, a 6F hockey stick guiding catheter (Cordis) was introduced and placed at the origin of the left coronary artery under fluoroscopic guidance, and a 3 mm diameter over-the-wire coronary catheter balloon (Cordis) was placed at the mid segment of the left anterior descending (LAD) below the origin of the first diagonal. The position of the catheter was verified by coronary angiography. An additional lidocaine bolus was also administered immediately before balloon inflation and deflation. The coronary artery was checked for patency by repeating the coronary angiography.

The second intervention was performed 30 ± 2 days later, when the pigs had developed a healed myocardial infarction. The animals were again sedated, anesthetized, and mechanically ventilated as in the first intervention. The femoral artery was catheterized and a 5F Millar micromanometer catheter (Millar Instruments) was advanced into the left ventricle (LV) to measure the LV pressure. A conventional ECG lead was continuously recorded. The thorax was opened through a midsternotomy and the heart was suspended in a pericardial cradle. An ultrasonic flow probe was carefully deployed around the aortic root to monitor the aortic blood flow (ABF).

### Electrocardiographic and hemodynamic parameters

In all cases we recorded the conventional ECG lead II (Nihon Kohden; Tokyo, Japan), the LV pressure (Nihon Kohden) and the ABF (Transonic Systems Inc., Ithaca, NY, US). All signals were digitized at 1 kHz (PowerLab with LabChart, ADInstruments) and stored for subsequent offline analysis. The first derivative of the LV pressure was also calculated.

### Myocardial electrical impedance

#### Theoretical background

The electrical impedance of the myocardium reflects an overall estimation of the intra- and extra-cellular resistances and the cell membrane capacitance (Gebhard et al., 1987; Kléber et al., 1987). Myocardial impedance has two components: tissue resistivity (ρ) and phase angle. Tissue resistivity quantifies the drop of voltage (V) amplitude for a given applied current (I). It was calculated from the relation R = k·ρ, where R is the in phase component of V with respect to I, and k is the cell constant of the electrode determined by measuring the electrical resistance of a 0.9% NaCl solution at 25°C, which affords a resistivity of 70 Ω·cm. Phase angle shift is related to the time delay between the voltage and current waves caused by the fact that biological tissues are not purely resistive.

#### Myocardial electrical impedance spectroscopy

**Figure 1:**
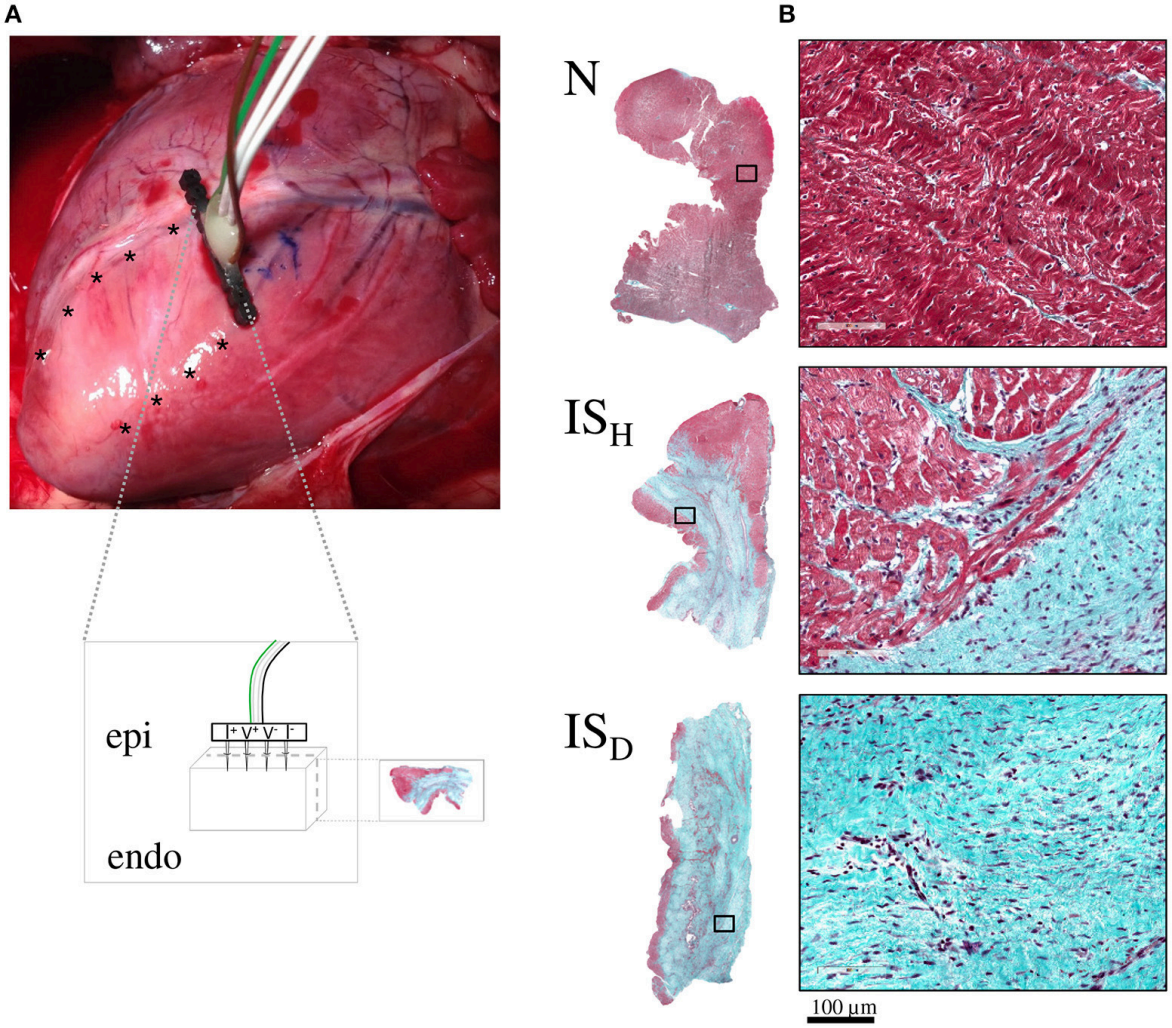
**Schematic representation of the experimental model. (A)** Photograph of a swine heart illustrating the location of the infarcted region in the anterior wall of the left ventricle and the sites where the impedance electrode was sequentially inserted (asterisks). The inset box depicts a schematic representation of the 4-needle impedance electrode (5 mm length spaced 1.27 mm, 0.4 mm diameter). **(B)** Representative microphotographs (at 1x and 20x magnification) of myocardial samples from normal zones (N) and from heterogeneous and dense infarct scar regions (IS_H_ and IS_D_, respectively) stained with Masson's trichrome.

Tissue impedance spectroscopy was measured as reported previously (Jorge et al., 2015). Briefly, a 4-needle electrode probe connected to a signal generation and acquisition system (PXI system, National Instruments) was inserted into the LV wall. The electrode was placed sequentially at different sites following a parallel linear array perpendicular to the trajectory of the LAD (Figure 1). Myocardial impedance spectroscopy was measured using current excitations consisting of a multisine signal (1 ms duration, 1 mA peak amplitude) of 26 frequencies logarithmically spaced in the range from 1 to 1000 kHz, leading up to 1000 spectra/s. The LV pressure, ABF and ECG signals were acquired simultaneously with the impedance signals using an additional digitizer card.

#### Systolic-diastolic myocardial electrical impedance

The impedance changes occurring throughout the cardiac cycle were analyzed. In each recording we measured the mean resistivity value, the amplitude of the phasic resistivity (maximum–minimum value) and the mean value of phase angle during the entire cardiac cycle. Thereafter, an analysis of the resistivity changes in relation to the LV pressure (LV pressure-impedance curves) was also performed. In addition, to determine the time relationship between the ECG and the systolic-diastolic myocardial resistivity, we measured the time interval between the R wave peak and the moment at which resistivity reached its maximum value.

### Anatomopathological examination

To assess the correlation between the degree of fibrosis in the infarcted region and the magnitude of local tissue impedance, we obtained myocardial samples in the same sites of impedance measurements. These sampling sites were marked with black ink after each myocardial impedance measurement to ensure an appropriate identification of the biopsies during tissue processing. The specimens were fixed in 10% buffered formalin, embedded in paraffin and sliced (4-μm thickness) perpendicularly to the epicardial surface up to the half of the sample block (Figure 1). After this, samples were stained with Masson's trichrome. All slides were digitized using an Aperio ScanScope Scanner (Aperio; CA) and the extent of the infarct scar was assessed by semi-automatic image quantification. Thus, the percentage of fibrosis was measured at 1.25X magnification using a custom-made Matlab algorithm (MathWorks; MA) which was based on color segmentation by K-means clustering (Wu et al., 2015). The percentage of fibrosis in each slide was calculated using the formula: 100 × (Area of fibrosis / Total slide area). According to the percentage of fibrosis the samples were categorized as proposed by Sivagangabalan et al. (2008); (1) healthy myocardium (N) (<10% of fibrosis); (2) heterogeneous infarct scar (IS_H_; 10–50% of fibrosis) and (3) dense infarct scar (IS_D_; >50% of fibrosis).

### Statistical analysis

Data were expressed as mean ± standard error of the mean (SEM). Differences in the study variables were assessed by the analysis of variance (ANOVA) with Bonferroni correction for *post-hoc* comparisons. Pearson's correlation between tissue fibrosis and impedance (resistivity and phase angle parameters) were calculated. A *P*-value < 0.05 was considered statistically significant. Statistical analyses were performed using the software SPSS v.22.0 (IBM SPSS Inc., Chicago, IL, USA).

## Results

**Figure 2:**
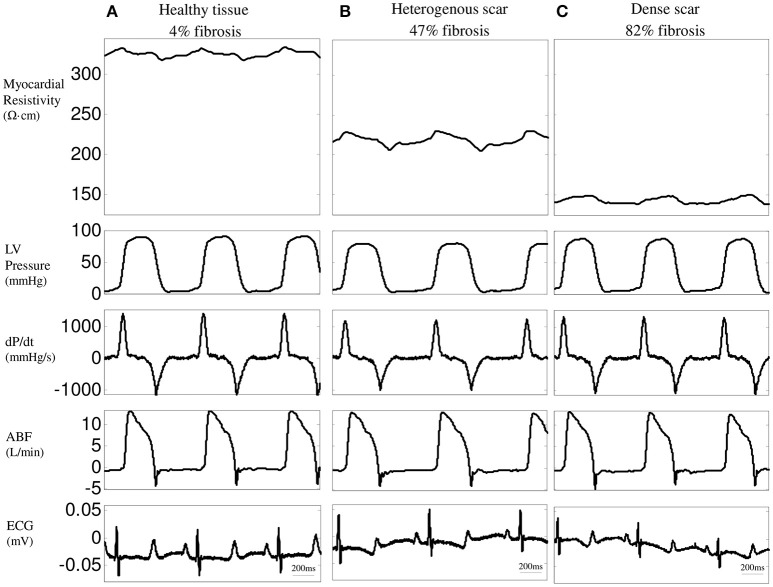
**Time-relationship between resistivity at 1 kHz, left ventricular (LV) pressure, LV dP/dt, aortic blood flow (ABF), and ECG in three representative LV regions: healthy tissue (A), heterogeneous (B) and dense infarct scar (C)**.

Two animal died during acute coronary occlusion due to irreversible ventricular fibrillation. The remaining animals (*n* = 6) completed their allotted follow up and were used for the study. As shown in Figure 1, 1 month after LAD occlusion there was a visible scar area in the anterior wall of the left ventricle in all animals. Local intramural myocardial impedance was recorded simultaneously with the physiological signals in a total number of 59 sites (Figure 2).

### Impedance spectroscopy

**Figure 3:**
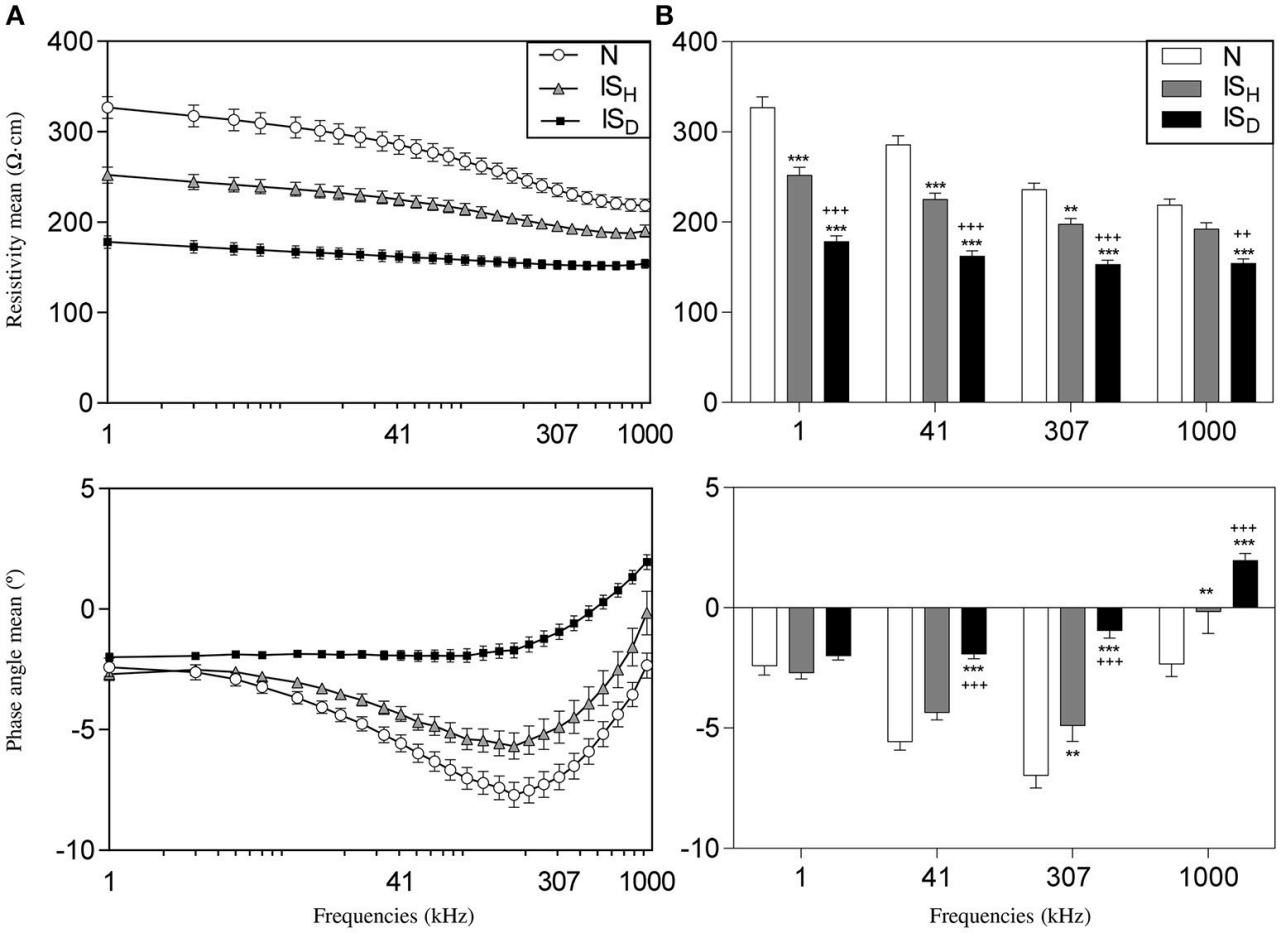
**Impedance spectroscopy of 1 month old myocardial infarction in the six studied pigs. (A)** Mean values of resistivity (top) and phase angle (bottom) of myocardial impedance at different excitation frequencies in normal (N), heterogeneous (IS_H_), and dense infarcted myocardial regions (IS_D_). **(B)** Mean resistivity (top) and phase angle (bottom) at 4 selected frequencies (1 kHz, 41 kHz, 307 kHz, and 1000 kHz). ^*^Significantly different from N (^**^ < 0.01, ^***^ < 0.001). ^+^Significantly different from IS_H_ (^++^ < 0.01, ^+++^ < 0.001).

Figure 3A shows the behavior of the mean values of resistivity and phase angle of normal and infarcted myocardium measured during the entire cardiac cycle at the 26 excitation frequencies studied in six pigs. Resistivity in normal myocardium decreased progressively at increasing excitation frequencies (from 327 ± 12 Ω·cm at 1 kHz to 218 ± 7 Ω·cm at 1000 kHz, *P* < 0.001). Moreover, phase angle spectrum showed a negative relaxation which was maximal at about 307 kHz. Regions with heterogeneous scar tissue (10–50% of fibrosis) showed a similar trend of changes (from 252 ± 9 Ω·cm at 1 kHz to 192 ± 7 Ω·cm at 1000 kHz, *P* < 0.001) although with a lower mean value (23% decrease at 1 kHz, *P* < 0.001) and a less marked phase angle relaxation. In contrast, sites with dense fibrosis presented an almost flat resistivity spectrum (178 ± 7 Ω·cm at 1 kHz and 154 ± 5 Ω·cm at 1000 kHz, *P* = ns) with lower mean resistivity values (46% reduction at 1 kHz, *P* < 0.001). Likewise, the phase angle relaxation vanished in the dense infarct scar.

Figure 3B illustrates the differences in the mean values of resistivity and phase angle at 4 selected frequencies (1, 41, 307, and 1000 kHz) in the different myocardial regions in six pigs. The excitation frequencies that better differentiated healthy myocardium, heterogeneous scar tissue and dense infarct scar were 1, 41, and 307 kHz for the resistivity and 307 and 1000 kHz for the phase angle.

### Correlation between local myocardial impedance and infarct fibrosis

**Figure 4:**
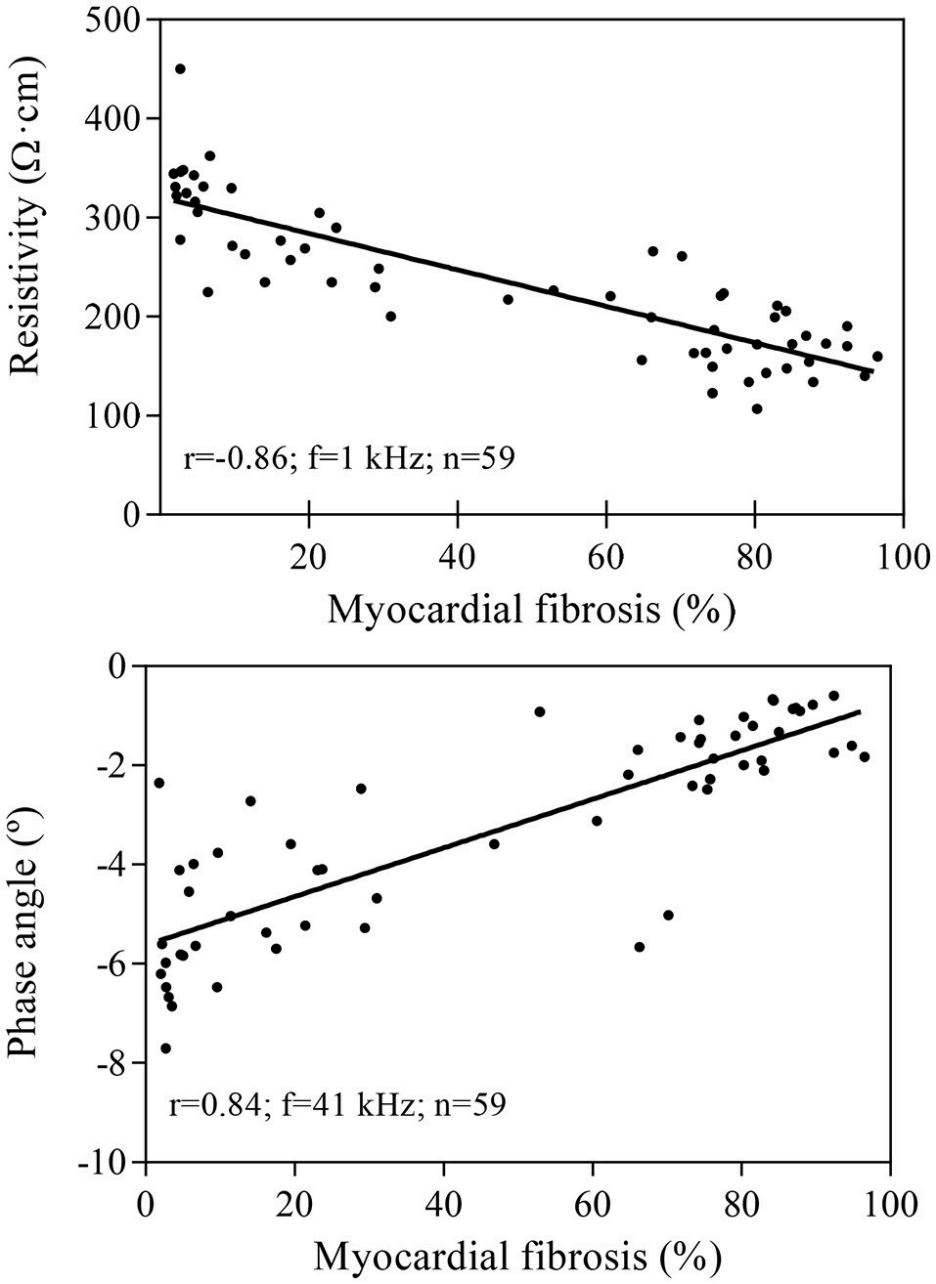
**Linear correlation between the local degree of myocardial fibrosis and myocardial resistivity (upper panel) or phase angle (lower panel) in 59 samples from six pigs**. Resistivity values are measured at 1 kHz and phase angle at 41 kHz.

As illustrated in Figure 4, higher degrees of fibrotic deposition in the infarcted region were associated with gradually lower mean values of local myocardial resistivity (*r* = −0.86 at 1 kHz, *P* < 0.001) and with a less negative phase angle deviation (*r* = 0.84 at 41 kHz, *P* < 0.001). Table 1 summarizes the values of the linear correlation coefficients at the four selected excitation frequencies of the studied spectrum (1, 41, 307, and 1000 kHz). Although a significant linear correlation existed between local tissue fibrosis and local bioimpedance parameters at most frequencies, the best discriminative frequencies were 1 kHz for myocardial resistivity and 41 kHz for phase angle.

**Table 1:** **Correlation coefficients (***r***) between electrical impedance and percentage of infarct fibrosis in 59 tissue samples from six pigs**.

	**Excitation frequency (kHz)**	***r***	***P***
Myocardial resistivity	1	−0.86	<0.001
	41	−0.85	<0.001
	307	−0.82	<0.001
	1000	−0.76	<0.001
Phase angle	1	0.19	= 0.15
	41	0.84	<0.001
	307	0.83	<0.001
	1000	0.67	<0.001

### Systolic-diastolic phasic changes in myocardial electrical resistivity

**Figure 5:**
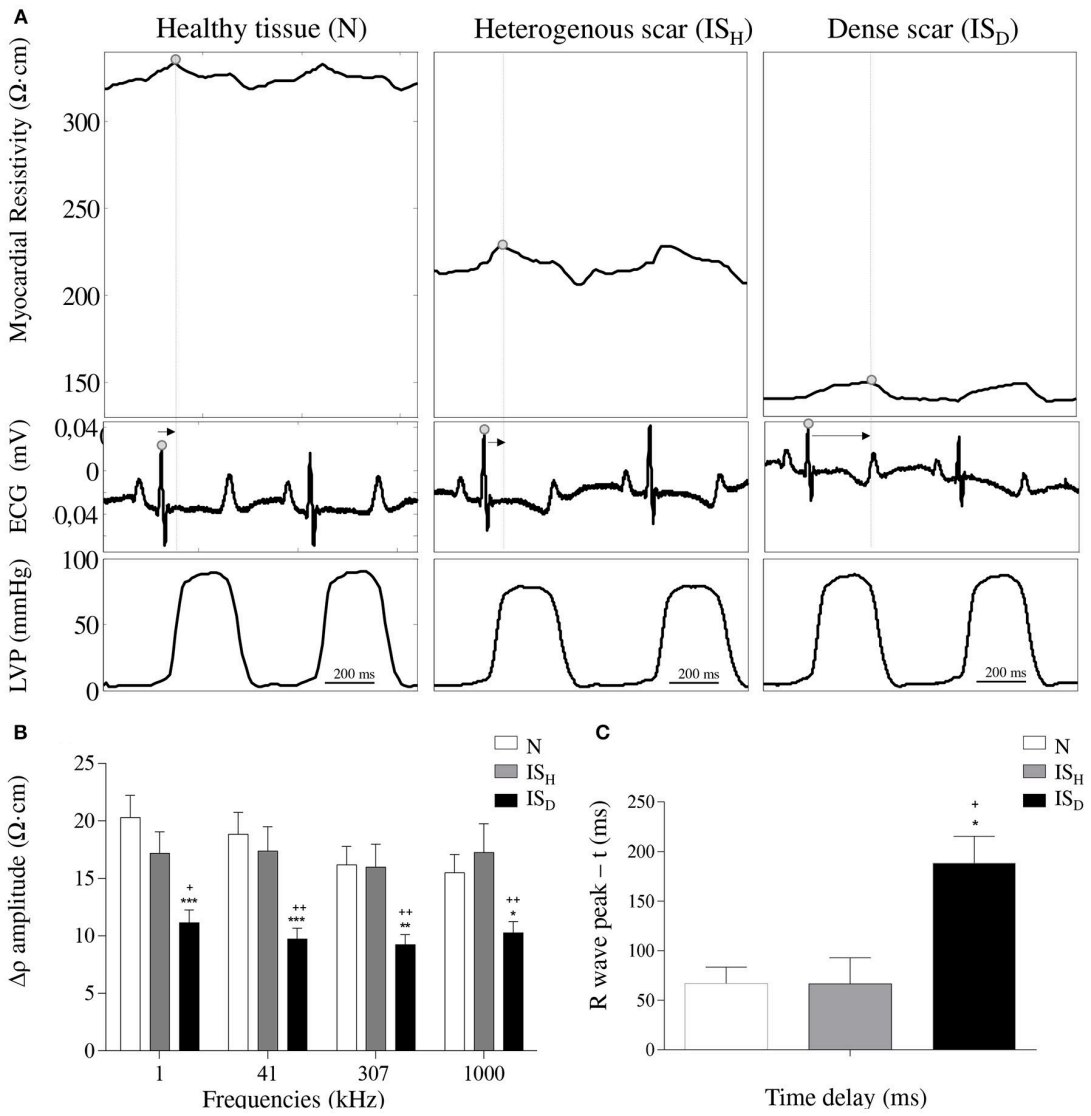
**Effects of infarct fibrotic content on myocardial resistivity curve. (A)** Systolic-diastolic myocardial resistivity waveform in healthy myocardial tissue (left), heterogeneous scar (middle) and dense scar tissue (right). Arrows indicate the time interval between the R wave peak and the max peak of the resistivity curve. **(B)** Mean values of the systolic-diastolic resistivity curve amplitude -Δρ- at 4 selected frequencies (1 kHz, 41 kHz, 307 kHz, 1000 kHz). **(C)** Mean temporal delays between the peak of the systolic-diastolic resistivity curve and its corresponding R wave peak of the ECG. Resistivity waveforms and delays are shown at 41 kHz. ^*^ Significantly different from N (^*^ < 0.05, ^**^ < 0.01, ^***^ < 0.001). ^+^Significantly different from IS_H_ (^+^ < 0.05, ^++^ < 0.01).

As shown in Figure 5, electrical resistivity of the normal myocardium depicted phasic changes during the cardiac cycle: a maximal value was attained during systole and a minimal value during diastole, leading to a mean amplitude of 18.1 ± 1.9 Ω·cm at 41 kHz. By contrast, infarct regions with dense fibrotic content showed a reduced systolic-diastolic impedance amplitude of 9.7 ± 0.9 Ω·cm, with only a small holosystolic displacement. Areas with less marked fibrotic density showed an intermediate pattern of systolic-diastolic impedance with an amplitude of 17.7 ± 1.9 Ω·cm.

Analysis of the temporal relationship between the myocardial impedance and the ECG showed that the systolic-diastolic resistivity curve of healthy myocardial regions presented its maximal peak shortly after the R wave. By contrast, Figure 5 illustrates that areas with densely infarcted myocardium were characterized by having its maximal resistivity peak beyond the R wave peak (188 ± 27 ms vs. 67 ± 16, *P* < 0.05) with an holosystolic plateau. The heterogeneous infarct scar zones attained their maximal resistivity peak at a delay similar to healthy myocardium.

### LV pressure-impedance curves during the cardiac cycle

**Figure 6:**
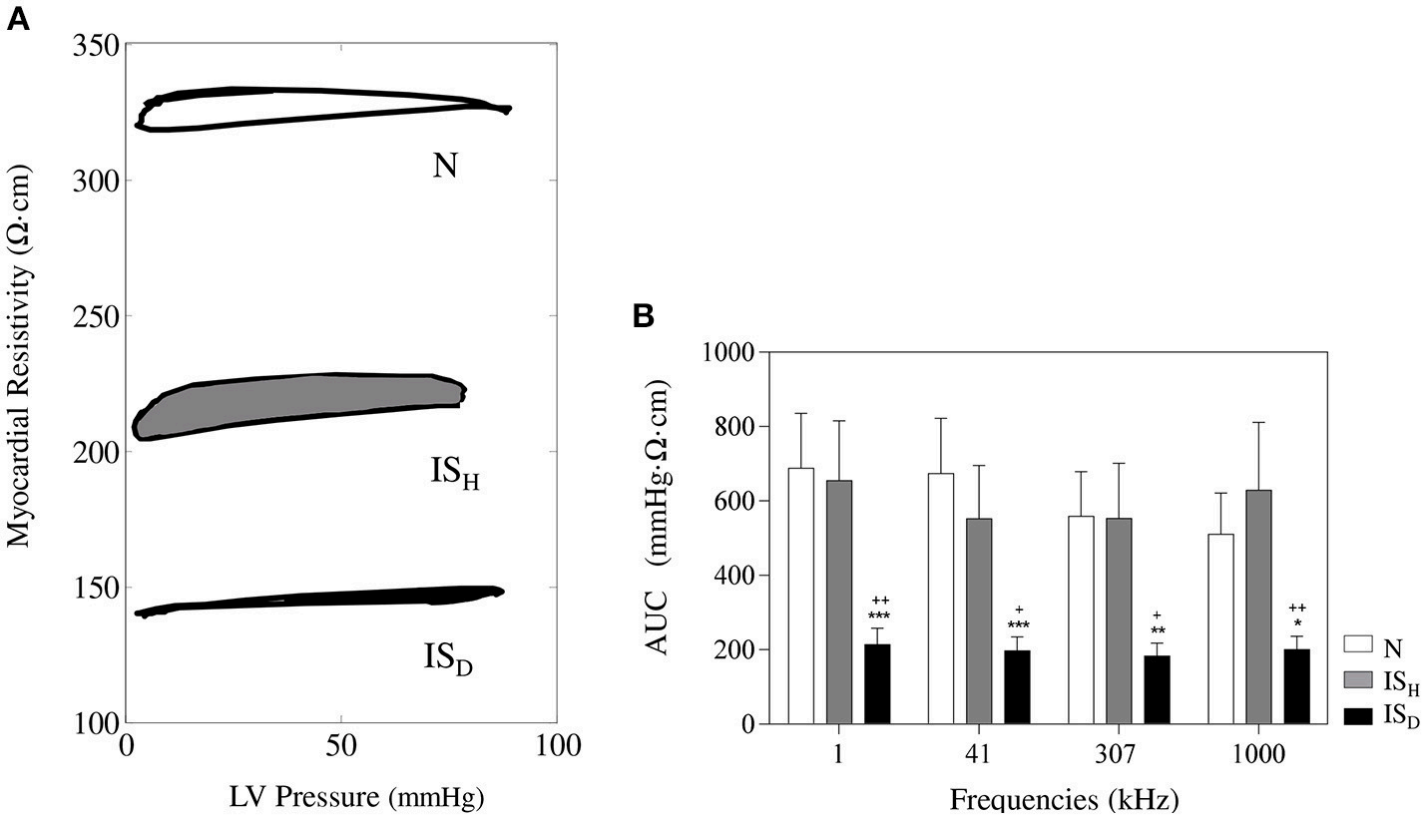
**Time relationship between the systolic-diastolic changes in myocardial impedance and left ventricular pressure. (A)** Representative LV pressure-impedance curves at sites with < 10% of fibrosis (white), 10–50% of fibrosis (gray) and >50% of fibrosis (black). **(B)** Mean values of the area under the curve at different current frequencies. ^*^Significantly different from set N (^*^ < 0.05, ^**^ < 0.01, ^***^ < 0.001). ^+^Significantly different from set IS_H_ (^+^ < 0.05, ^++^ < 0.01).

The temporal relationship between the whole cardiac mechanical activity and the concurrent changes in regional myocardial impedance can be better analyzed by constructing the LV pressure-impedance (LVPI) curves. As illustrated in Figure 6, dense infarcted zones showed an area under the LVPI curves (AUC) smaller than the heterogeneous scar tissue or the healthy myocardium (IS_D_: 214.7 ± 44.2 mmHg·Ω·cm, IS_H_: 655.4 ± 160.7 mmHg·Ω·cm, N: 665.5 ± 137.8 mmHg·Ω·cm; *p* < 0.05 IS_D_ vs. IS_H_ and *p* < 0.01 IS_D_ vs. N). These changes were comparable among the different studied frequencies.

## Discussion

### Main findings

This study reveals that the degree of fibrotic deposition in the infarcted myocardium is a direct determinant of both the local decrease in tissue impedance and the attenuation of systolic-diastolic impedance oscillations.

### Electrical impedance of normal and infarcted myocardium

Previous experimental studies in models of transmural healed myocardial infarction induced by permanent coronary occlusion have consistently found low electrical resistivity values in the necrotic scar (Fallert et al., 1993; Warren et al., 2000; Wolf et al., 2001; Salazar et al., 2004). However, the prolonged time required for measuring the impedance spectrum in these studies prevented knowing the time course of myocardial impedance during the whole cardiac cycle. A recent refinement of the bioimpedance method based on fast broadband EIS permitted to characterize the changes in myocardial impedance induced during the cardiac cycle under normal and acute ischemic conditions in the *in situ* porcine heart (Sanchez et al., 2011; Jorge et al., 2015). In that model the normal myocardium depicted a biphasic resistivity pattern that turned to a bell-shaped morphology with an increased mean value following coronary occlusion.

The moment at which the high-resistivity, biphasic bioimpedance pattern observed in the acute phase of ischemia evolves to a low-resistivity and flat systolic-diastolic pattern in the healed infarcted tissue is not well defined. Previous studies in sheep showed the 200% increase in myocardial impedance after 4 h of permanent coronary occlusion that turned to a 60% decrease 1 week later, and this was maintained 6 weeks after (Fallert et al., 1993). The mechanisms implicated in the progressive reduction of tissue resistivity during infarct healing are likely related to the sequential occurrence of interstitial edema, loss of myocyte content, and collagen and fat deposition in the ischemic region (Fallert et al., 1993; Schwartzman et al., 1999; Del Rio et al., 2008; Farraha et al., 2014). These structural derangements will be less marked in infarction areas with surviving myocardial cells and therefore could be detected by bioimpedance techniques. In 4 sheep with 60-days old anterior myocardial infarction induced by permanent ligature of the LAD coronary artery, epicardial impedance measured with the 4-electrode technique at 1 kHz was lower in the visually defined dense infarction than in the border zone or in the healthy myocardium (Schwartzman et al., 1999). Using a 3 h LAD occlusion-reperfusion model in three sheep it has been reported that areas with greater myocardial injury (<50% viability), assessed by histologic examination, had greatest attenuation of impedance magnitude compared with areas with >50% viability in a 5 weeks-myocardial infarction (Farraha et al., 2014).

As a novelty, our data show a robust correlation between the local degree of fibrosis and the values of resistivity and phase angle, based on a quantitative site-by-site analysis. Indeed, dense scar areas exhibited the lowest resistivity values and the sites with intermediate degree of fibrosis showed transitional values. Moreover, we found that tissue viability can also be assessed by changes in the amplitude of systolic-diastolic resistivity and by the analysis of the temporal delay between phasic myocardial resistivity and the ECG. We recently proposed that the amplitude of the impedance curve is modulated by the mechanical cardiac activity thus the flat resistivity curve of the dense scar could be in part due to the local severe dyskinesis (Helle-Valle et al., 2009; Garcia-Sanchez et al., 2015). In addition, the drop of the AUC supports the hypothesis that the LVPI curves would allow differentiating the segments able to generate active force (healthy tissue) from those that are dyskinetic (necrotic tissue) and therefore mechanically passive (Jorge et al., 2015). The AUC reduction is caused by two main factors: (1) the low-amplitude of the impedance phasic changes and (2) the delayed occurrence of the resistivity peaks. In fact, the significant delay between the peaks of systolic-diastolic resistivity of the infarct scar and the physiological signals could also be a consequence of the passive movement of this tissue, reinforcing the hypothesis that the analysis of the systolic-diastolic myocardial impedance curves may be useful to recognize the infarct scar.

### Impedance spectroscopy

The resistivity and phase angle values are highly dependent on the frequency of the applied current: the low frequency currents flow preferentially through the extracellular space, whereas the high frequency currents can pass through the cell membrane capacitance and therefore could flow through both the intracellular and extracellular compartments. Since the infarcted tissue is characterized by loss of myocardial cell structures and replacement of extracellular space by collagen deposition, the applied current frequency will have a negligible influence on the values of resistivity and phase angle. This concept was confirmed in previous studies (Cinca et al., 1998; Warren et al., 2000; Salazar et al., 2004) and also in the present investigation. However, as a novel finding our data revealed that heterogeneous areas of infarction containing viable myocytes do show different frequency related patterns (Foster and Schwan, 1989). Interestingly, we found that the amplitude of systolic-diastolic resistivity either in normal or infarcted tissues does not change with the applied current frequency. Thus, suggesting that the amplitude variations in local resistivity in normal and infarcted tissue are more dependent on the strength of local mechanical activity.

### Clinical implications

Our data represent the proof-of-concept that myocardial impedance can identify the degree of fibrosis, and therefore the extent of the infarct scar. Theoretically, fibrotic myocardial infiltration other than that of ischemic origin (i.e., infiltrative diseases, myocarditis, hypertension) could also be detected by means of bioimpedance methods in atrial or ventricular level. Thus, recognition of local fibrosis by endocardial catheter mapping would improve the yielding of radiofrequency catheter ablation of either atrial or ventricular arrhythmias.

### Study limitations

The impedance measurements described in this study were obtained with an intramural 4-needle electrode probe in the open-chest swine model in order to achieve optimal recording sensitivity and accessibility to tissue samples. Due to the thinning of the left ventricle in the sites of infarction we cannot entirely exclude that the impedance spectrum at those site could be more flat due to the short distance between the blood volume in the ventricle and the tip of the 4-needle electrode. Theoretically, this could affect the decrease in the systolic-diastolic impedance values. However, *in vitro* calibration measurements using the 4-needle electrode probe in contact with blood showed much lower impedance values with completely flat impedance spectrum.

On the other hand, this method is an invasive approach precluding direct clinical use until an intracavitary approach based on endocardial contact electrocatheter probe is developed. We have previously reported in the swine model, that the changes in myocardial resistivity induced by coronary occlusion can be successfully detected with an electrocatheter based approach in the closed chest (Warren et al., 2000). In the canine model, other authors have been able to delineate the borders of infarct scar using endocardial catheter impedance mapping (Wolf et al., 2001).

## Conclusions

As compared with the normal myocardium, the local impedance of healed myocardial infarction tissue shows lower mean values and depressed systolic-diastolic oscillations. These changes have their maximal expression in infarcted regions with greater fibrotic content. Once this new bioimpedance method can be implemented in a catheter-based device it might have potential clinical applicability in the detection of fibrotic tissue to guide the ablation of atrial or ventricular arrhythmias.

## Author contributions

JC, RB, and JR conception and design of research; GA, EJ, TG, RB, JR, and JC performed experiments; GA and EJ analyzed data; GA, EJ, and JC interpreted results of experiments; GA and EJ prepared figures; GA, EJ, and JC drafted manuscript; GA, EJ, TG, RB, JR, and JC edited and revised manuscript. GA, EJ, TG, RB, JR, and JC approved final version of manuscript.

### Conflict of interest statement

The authors declare that the research was conducted in the absence of any commercial or financial relationships that could be construed as a potential conflict of interest.
